# A Systematic Review on Spinal Asymmetries in Case Studies of Unilateral Nephroptosis from a Viscerosomatic Point of View

**DOI:** 10.3390/healthcare10122422

**Published:** 2022-11-30

**Authors:** Ángel Oliva-Pascual-Vaca, María José Castillo-Cañuelo, Jesús Oliva-Pascual-Vaca, María Pérez-Montalbán, Francisco Javier Ordonez, José Antonio Martínez-Fernández

**Affiliations:** 1 Department of Physiotherapy, Universidad de Sevilla, 41009 Sevilla, Spain; 2 Escuela de Osteopatía de Madrid, 28807 Madrid, Spain; 3 Instituto de Biomedicina de Sevilla (IBIS), 41013 Sevilla, Spain; 4 Escuela Universitaria Francisco Maldonado, 41640 Osuna, Spain; 5 School of Sports Medicine, University of Cadiz, 11003 Cadiz, Spain

**Keywords:** physical examination, osteopathic medicine, posture, diagnostic imaging, nephroptosis, scoliosis

## Abstract

The assessment of posture and asymmetries is common in musculoskeletal clinical practice, and correction is a frequent goal. In this setting, posture and asymmetries are usually interpreted in terms of musculoskeletal issues. This study aimed to evaluate spinal asymmetries in case studies of unilateral nephroptosis. A systematic review was performed using PubMed, CINAHL, Scopus and Web of Science. We included case reports and case series of nephroptotic patients which showed diagnostic imaging that allowed us to assess the presence of spinal asymmetries in the frontal plane. The methodological quality of the selected studies was assessed by using Case Report (CARE) checklist. Nineteen studies were included, with a total number of 78 reported patients (69 women) ranging 22 to 44 years old (mean: 29). Only one patient presented with medial nephroptosis, while the rest presented with caudal migration. Ninety-one percent of the cases affected to the right kidney. All cases but two showed homolateral flank closure (lower rib descent, iliac crest raise and/or homolateral side-bending). The correction of nephroptosis, either by supine position or surgical treatment, removed asymmetries in some cases while other cases improved only partly. Manual therapists must consider visceral implications while assessing body posture. Further, since the most common symptom of nephroptosis is loin pain, and it has been claimed that loin pain is underdiagnosed, manual therapists should consider its potential presence during clinical practice. Finally, being that nephroptosis shares several features with idiopathic lumbar scoliosis (type of patient, postural adaptation), more research is needed regarding any possible relation between them.

## 1. Introduction

Manual therapists frequently analyse the posture of the patient during diagnosis and evaluation, and the finding of asymmetries is commonly addressed, such as, for instance, the height of the shoulders or the iliac crests during visual inspection or palpation [1]. Furthermore, while managing patients, improvement of posture and asymmetries is a frequent goal [1,2].

Body posture can have repercussions on the viscerae in several ways, influencing on issues such as tachycardia or esophageal peristalsis [3]. In the case of the kidney, several studies show the effects of posture in renal status, the development of pathology, the potential of using postural therapies as treatments for certain kidney disorders and even the best position while using extracorporeal shockwave lithotripsy [4,5,6,7,8,9,10]. In the same way, the consequences of renal compromise may affect postural balance through systemic affectation [11].

The influence of body posture on pain symptoms in nephroptosis has also been described. This condition is defined as the kidney descending two vertebral bodies (or more than 5 centimeters) when going from laying to standing, allowing the return of the kidney to its anatomical position when turning back to supine [12]. A history of loin or flank pain relieved by lying down is the most common symptom among patients with pathologic nephroptosis [13]. Population incidence of this abnormal mobility is difficult to establish because most cases are asymptomatic during a person’s whole life or most of it. However, it is known that 70% of cases of ptosis affect the right kidney, 10% the left kidney and 20% are bilateral. The male/female ratio is approximately 3:100 for radiologically detected cases [14], being more common in young, slim women [15].

Nephroptosis may trigger several consequences, such as ureteral obstruction and hydronephrosis, ischemia due to elongation, narrowing or torsion of the renal artery, and kidney occlusion. Further, this occlusion may produce venous stasis, traction and visceral nerves stimulation in the hilum region and thus generate symptoms related to these consequences [16]. Only 10–20% of the cases are symptomatic [16].

One of the functions of the musculature is protecting the integrity of body tissues, obviously by means of contraction. This contraction may suppose a change in body posture, which is known as antalgic posture [17]. Visceral afferents trigger the activation of somatic efferents on muscles, with the aim of achieving sustained contraction [18]. The posture adopted to protect a nerve root in the case of a lumbar disc herniation is a well-known example [17]. It has been shown that this protective contraction appears before the sensation of pain takes place, thus constituting antalgic activity [19,20,21,22,23]. Similar to the muscular activity that protects neural tissues, muscles also contract with the aim of protecting visceral structures. For instance, this activity occurs in the abdominal wall during appendicitis. As well, emetic contractions also occur in an attempt to protect the integrity of the subject during visceral problems. Regarding kidneys, that muscle activity has been experimentally demonstrated through unilateral artificial ureteric calculosis in rats, which triggered contraction in the ipsilateral oblique musculature [24]. In the clinical setting, it is well known that patients suffering from renal colic may adopt a particular posture [25].

To our knowledge, the analysis of posture in visceral disorders has not been particularly developed to date in the case of nephroptosis. Therefore, the aim of this study is to evaluate the postural modification observed in subjects with renal ptosis.

## 2. Materials and Methods

This systematic review has been performed according to the Preferred Reporting Item for Systematic Reviews and Meta-Analyses (PRISMA) guidelines. It was registered in the International Prospective Register of Systematic Reviews (PROSPERO), with registration number CRD42022321551.

### 2.1. Data Sources and Search Strategy

A systematic search of PubMed, CINAHL, Scopus and Web of Science was performed between 22 and 25 March 2022 with no limits on dates of publications. The search used the key terms *renal*, *kidney*, *ptosis*, *nephroptosis* and nephr*. In the first two databases, the case report filter was used. In Scopus and Web of Science, the terms “case report*” and “*case series*” were also used. The search was carried out by two independent reviewers while, in case of any disagreement, a third researcher was consulted.

### 2.2. Study Selection

We included in this review any article about case report or cases series, published in English or Spanish, showing diagnostic imaging which allows us to analyse the spinal asymmetries in the frontal plane in subjects suffering from unilateral nephroptosis. In the case of bilateral ptosis, a higher displacement in one kidney had to be present in order to be included. Also, studies were excluded in the case of children who had not experienced standing posture (younger than 18 months), pregnant individuals, individuals whose kidney ptosis was secondary to toxic expositions, pelvic prolapse, or individuals who also had heart, neurological, mitochondrial, metabolic or congenital disease. Similarly, cases presenting transplant, systemic infectious disease, tumours or musculoskeletal malformations were also excluded. Two independent researchers performed the study selection, while a third one resolved any disagreement.

### 2.3. Methodological Quality

Case Report (CARE) checklist was used in order to assess the quality of the reviewed studies. This was developed to increase the accuracy, transparency and usefulness of case reports [26]. The analysis of the cases was also independently performed by the same researchers who participated in the study selection. The same third researcher solved any disagreement.

### 2.4. Data Extraction

Once the studies were selected, the authors independently assessed each study and collected certain data: demographic data, clinical history, symptoms, clinical findings, diagnosis, postural radiological findings, therapeutic intervention and outcomes.

## 3. Results

**Figure 1:**
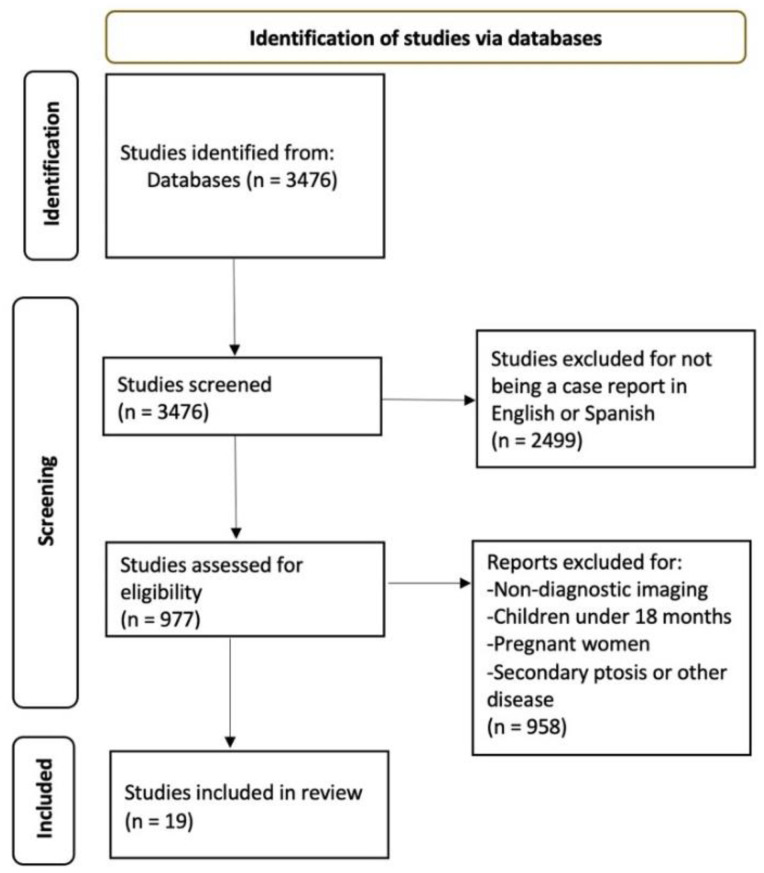
Flow chart of the selection process (PRISMA).

From the search strategies carried out in the different databases, a total of 3476 articles were obtained. The criteria of being case studies in English or Spanish was applied to obtain 977 studies, of which 958 were discarded when considering the exclusion criteria and the aim of the study. After reading the full text, 19 articles were included in the systematic review. Figure 1 shows the flow chart summarising the selection process.

All the selected articles studied only one case, except five of them, which presented case series [27,28,29,30,31] (Table 1). However, in two of these studies, only the first case was included, because the rest did not meet the established selection criteria [30,31]. The assessment of the methodological quality is shown in Appendix A. The studies have total scores ranging from 5/30 to 18/30 according to the CARE checklist.

A total of 78 people with nephroptosis was included. A total of 94.74% of the cases were women, with only one of the studies presenting a case series included men (nine men) [27]. The mean age was 29 years, ranging from 22 to 44 years. Only one case presented medial renal ptosis [32], while all of the rest presented caudal ptosis. In addition, the right kidney was the injured one in 91.03% of the cases, with only seven subjects in one study [27] suffering from left-sided ptosis.

Pain was reported in 16 of the 19 articles included in the review. This pain was located in the abdominal area or ipsilateral flank in 84.62% of cases [27,29,30,31,33,34,35,36,37,38,39,40]. Pain could also affect the spine, involving the lumbar area [41] and the right spinal area [42], and even follow a radiating distribution from the abdominal and lumbar areas to the groin [43,44]. Regarding medical history, one subject with joint laxity [43], one subject with lumbar hernia [42] and one subject with L3-L4 spinal fusion [31] were included.

On physical examination, three studies [30,33,43] reported finding a palpable mass in the abdominal or right flank area with the subjects in a standing position. In contrast, two studies [27,45] observed this finding while the subjects were in the supine position.

Analysis of the pre-intervention imaging tests shows the pathological descent of the kidney in all cases in the standing position, even to the level of the sacrum [33] (Table 2). In those cases where an intervention was performed and post-intervention images were available, an improvement in the position could be observed [27,28,29,31,33,34,42], as shown in Table 3.

In terms of postural attitude, homolateral flank closure to the side of the ptotic kidney was observed in all the cases included in the review, with the exception of the patient in the study by O’Reilly et al. [30] and the second case of the article by Jungling et al. [33]. This happens especially in standing, and it usually decreases or disappears after treatment, although not in all the cases [27,28,29,31,33,34,42]. The protective attitude was generated by one or more of the following postural adaptations: homolateral last rib lowering, homolateral iliac crest raising and/or homolateral side-bending. It should be noted that this postural attitude was also observed in the patient suffering from medial ptosis [32].

Finally, only three cases did not receive therapeutic intervention [40,41,45]. Most participants underwent nephropexy to restore renal cephalic position and eliminate or improve symptom intensity. Conservative [39,42,43] and psychiatric [43,44] treatments were also applied, and physiotherapy was considered in one case [34]. Improvement was visible in all cases except in two subjects [27].

## 4. Discussion

The aim of our study was to evaluate postural modifications in subjects with nephroptosis. According to our results, nephroptosis has shown to generate spinal adaptations, with a protective posture by means of a flank closure, achieved by lowering of the homolateral lower rib, iliac crest raising and/or homolateral side-bending, with multiple vertebral asymmetries. Our results also show that, in some cases, postural asymmetries disappear when the ptosis is corrected (in supine position [31,35,36,38,39,40,43,44,45] or after surgical treatment [27,29,31,34,42], but in some cases they only improve partly [28,33], probably as a consequence of a long-term attitude, long-term muscular activity and shortening.

In respect to manual therapists, our study acts as a reminder that postural asymmetries should not be just considered somatic issues. As previously explained, muscles are recruited in order to prevent damage in important tissues with a protective function [20,21,22]. Thus, the body achieves an antalgic or lower-antalgic posture [17]. Our review covers diagnosed patients, suffering mainly pathologic nephroptosis [13], living with the effects of several symptoms. However, as previously exposed, only 10% to 20% of nephroptosis are symptomatic [14], and nephroptosis is frequently misdiagnosed and underreported [13,34,46]. It must be considered that asymptomatic people may be asymptomatic thanks to muscle hypertonus, since muscular activity can allow the absence of symptoms by antalgic posture. In the case of kidney ptosis, most patients are asymptomatic [46], and some of those asymptomatic subjects might be showing antalgic posture and asymmetries. Further, the degree of kidney ptosis needs to be higher than five centimetres in order to be considered nephroptosis. Subjects with a smaller renal descent might be adapted by minor postural changes.

It is generally considered that visceral pain has nothing to do with body posture and movement. However, our study does not support that assumption. Subjects suffering from nephroptosis improve with lying down and get worse while standing, walking or running [13]. Of course, organs are stressed by gravity, such as when it happens in visceral (and renal) ptosis. Similarly, musculoskeletal disorders mimicking spleen pain can be modified by lying still, coughing or during physical activity [47,48]. Even pain during coronary issues can disappear in some cases by the adoption of certain postures [49].

Regarding pain, it has been exposed that pain is usually the most important symptom in nephroptosis, either felt in the lumbar area [28], loin [50], abdominal or flank [44]. To avoid misdiagnosis related to musculoskeletal issues, nephroptosis must be considered, since a mechanical component is also involved in nephroptosis [13,27,35]. In our sample, five cases had lumbar or loin pain [30,31,42,44]. Further, another patient felt groin pain, which also can be misdiagnosed as a musculoskeletal issue [44]. It is also interesting to note that nephroptosis pain can be misdiagnosed as psychosomatic [43].

The presence of nephroptosis has been linked to direct trauma related to sports, childbirth and caesarean section but also to weight loss [51,52,53] due to the loss of the supporting perirenal and pararenal fat [54]. Some cases in our sample showed diminished kidney support, either by caesarean section [40], low body mass index [27] or hysterectomy [30]. In this sense of losing support, laxity [43] might not only affect the stability of joints but also that of the organs, allowing visceroptosis [55]. Also, abdominal pressure indirectly contributes to maintain renal position [56]. Thus, one patient was prescribed abdominal wall strengthening exercises [43].

Scoliosis is a three-dimensional anatomical deformity of the spine in which there is a lateral displacement in the frontal plane, a spinal rotation in the horizontal plane and a modification of the physiological curves in the lateral plane [57] greater than 10° of angulation (according to Cobb’s method) [58]. At the lumbar level, the most common pattern is the right concavity, accounting for 70% of cases [59,60]. Curiously, the prevalence of right nephroptosis is 70% [13,14], and our study shows that these patients present a closure of the right flank. On the other hand, idiopathic scoliosis accounts for 75% to 80% of all case of scoliosis [61]. In addition, idiopathic scoliosis has a high incidence in adolescents and in females, with 70% of cases [62,63,64], and the risk of developing scoliosis is known to be increased in individuals who are underweight or have suffered an excessive weight fluctuation [65]. Similarly, nephroptosis is much more common in young, slim women [14,15]. Given the similarities between the two pathologies, it seems that the presence of nephroptosis should be ruled out during the assessment of idiopathic scoliosis. Our review supports these common points between nephroptosis and scoliosis since the sample in our study was constituted mainly by women with right nephroptosis producing a closure of the right flank.

It is interesting to note that a recent systematic review has shown that manual therapy improves forward head posture, thoracic kyphosis and pelvic alignment, but it does not improve scoliosis [2]. Perhaps the viscerosomatic influence might help to explain this fact, either because the therapist only treats musculoskeletal tissues or because the visceral disorder is so severe that it cannot be improved manually.

Thus, according to our results, manual therapists should consider the possibility of an underlying primary visceral disorder during postural analysis, at least in relation to nephroptosis and asymmetries in the frontal plane (flank closure). Further, while evaluating patients suffering groin, abdominal, flank or low back pain, manual therapists should have a higher suspicion of nephroptosis involvement when any of these features are present: female young or middle-aged patients with pain in the right side, slim or with a history of weight loss, visceral support deficit, scoliosis, and/or worse symptoms in upright positions that improve in decubitus. However, more research is needed to describe, in a more comprehensive way, possible postural implications of visceral disorders affecting not only the kidney, but also other organs. 

With regard to the limitations of the present review, it should be noted that we have reviewed case reports and case series, and this kind of studies are at the lower level of scientific evidence according to the methodological design. In relation to the methodological quality, it should be noted that, within the CARE checklist, scores below half were obtained. Therefore, studies of higher methodological quality are needed in the future to confirm the findings. Further, many studies were excluded during the search process because they showed no diagnostic imaging allowing to analyse the spinal asymmetries in the frontal plane. Besides, several studies do not show complete spinal images. On the other hand, pre- and post-intervention images in the supine and standing positions were only available in one case [31]. As well, the case series did not include diagnostic images of all participating subjects. Finally, we only evaluated asymmetries in the frontal plane.

## 5. Conclusions

Nephroptosis is related to a postural adaptation in the frontal plane, which increases when standing upright, and the symptoms usually decrease after treatment. This postural attitude (flank closure) is compatible with an antalgic position to protect the body structures affected by nephroptosis. Several features of nephroptosis are similar to those of idiopathic scoliosis; therefore, nephroptosis should be ruled out during the scoliosis assessment.

## Figures and Tables

**Table 1:** General (non-postural) data of the study sample.

Studies	Subjects	Medical History	Symptoms	Clinical Findings	Diagnosis	Intervention	Follow-Up
**Khan et al.** **[27]**	42 Female 9 Male 18–75 years		Ipsilateral flank pain aggravated by walking. 13/51 moving mass. 9/51 lithiasis in the ptotic kidney.	PE: depression of abdominal mass in 13/51 in the SUP. Ultrasound: right renal descent. IU: shows a shortened and descended ureter.	Right 44/51 left 7/51 nephroptosis	Nephropexy	2 months: kidney well positioned. 3.5 years on average: 3/51 with symptoms, 2/51 remain ptosis.
**Machado Bairo et al. [28]**	6 Female 34.3 average years	1/6 appendectomy	Long symptomatic course. No lithiasis.	IU: superior renal descent when UP versus SUP. Renal scintigraphy: 5/6 obstructive pattern and 1/6 ischaemia when UP.	Right nephroptosis	Right nephropexy	21,6 months: 5/6 with no pain, no obstructive pattern, correct position. 1/6 less pain, no ischaemia and reduced descent.
**Lezrek et al.** **[29]**	4 Female 30.5 average years		Abdominal pain on the right side when UP for a prolonged time.	Ultrasound: right renal descent when UP. IU: inclination with shortening of the ureter when UP.	Right nephroptosis	Right nephropexy	28 average months: 4/4 no symptoms. Ultrasound and IU kidney higher when UP.
**O’Reilly et al.** **[30]**	1 Female 30 years		Right abdominal pain and presence of a right paraumbilical mass.	PE: palpable right abdominal mass, visible and painful when UP. IU: descended right kidney. Renography: abnormal mobility of the right kidney when UP.	Right nephroptosis	Right nephropexy	Renography normal in both positions. No symptoms.
**Marcovich et al.** **[31]**	1 Female 38 years	L3-4 spinal fusion. Total hysterectomy.	Right flank pain worse when UP, relief in SUP.	IU: in any position rotation and right nephroptosis. Diuretic renogram: 30% right renal function without obstruction in the SUP.	Right nephroptosis	Right nephropexy	6 weeks: renal ptosis resolution. 29 weeks: no pain.
**Leong et al.** **[32]**	1 Female 34 years		Intermittent right upper abdominal protrusion. No abdominal pain.	CT: medial deviation of the right kidney with moderate hidronephrosis. IU: minimal deviation of the right ureter when UP.	Right medial nephroptosis	Right nephropexy	6 months: normal anatomical position of the right kidney.
**Jungling** **et al. 1º** **[33]**	1 Female 26 years		Recurrent dull pain in the right lower AQ. Increased pain when UP, relief in SUP.	PE: mobile mass on the right flank when UP. IU: ptotic kidney with hidronephrosis when UP.	Right nephroptosis	Nephropexy and right pyeloplasty	IU when UP normal and complete resolution of symptoms (6 weeks).
**Jungling** **et al. 2º** **[33]**	1 Female 31 years		Dull, intermittent pain in the right lower AQ. Increased pain when UP, relief in SUP.	PE: mobile mass on the right flank when UP. IU: right kidney descended and rotated when UP. Arteriography: two anomalously located renal arteries.	Right nephroptosis	Right nephropexy	No pain. IU no description.
**Tunc et al.** **[34]**	1 Female 34 years		Right flank pain which decreases in the SUP. Feeling of falling into the abdomen when UP.	IU and abdominopelvic ultrasound: both positions show minimal dilatation of the collecting system, shortening of the right ureter and nephroptosis. VAS: 10/10.	Right nephroptosis	Pain unit and physiotherapy. Right nephropexy.	VAS 1/10. 60 days: IU cephalic position right kidney. 210 days: IU without complications.
**Boylu et al. [35]**	1 Female 22 years		Persistent non-specific right flank pain when UP and relief in the SUP.	Ultrasound: moderate to severe right hidronephrosis without dilatation of the ureter. IU and fluoroscopy: right renal descent when UP.	Right nephroptosis and right UPJO	Simultaneous right pyeloplasty and nephropexy	3 weeks: unobstructed
**Baldassarre et al.** **[36]**	1 Female 34 years		Right flank pain when UP.	Renal ultrasound and IU: 5 to 6 cm descent of the right kidney when UP.	Right nephroptosis	Right nephropexy	2 months: slight pyelectasis and clinical improvement. 6 months: improvement of ptosis
**Sood et al.** **[37]**	1 Female 28 years		Intermittent right abdominal pain that worsens when UP.	IU: 6 cm craniocaudal migration of the right kidney when UP.	Right nephroptosis	Right nephropexy	4 weeks: symptoms improvement, not pain. 6 years: decrease of symptoms.
**Wroclawski et al. ** **[38]**	1 Female 29 years		Pain on right side.	PE: mobile and palpable right kidney. Ultrasound, IU and renal scintigraphy: confirms right renal ptosis when UP.	Right nephroptosis	Right nephropexy	1 month: symptoms improvement, normal function and appropriate position.
**Hua Chan et al. ** **[39]**	1 Female 40 years		Long-term right-sided pain.	PE: soft abdomen without pain, kidneys not votable. Intravenous pyelography: descent of the right kidney when UP.	Right nephroptosis	Conservative TX and clinical follow-up	
**Yoshida et al.** **[40]**	1 Female 38 years	Caesarean section at the age of 26. Hyperaldosteronism.	Recurrent abdominal pain of more than 12 months’ duration.	PE: no acute findings. LT: slight elevation of C-reactive protein. Ultrasound: showed the positional changes of the right kidney.	Right nephroptosis		
**Carola et al.** **[41]**	1 Female 26 years		Lower back pain and discomfort in the right lower AQ.	LT: low creatinine and urine creatinine values. Renal scintigraphy and IU: renal ptosis.	Right nephroptosis		
**Matsuda et al. ** **[42]**	1 Female 44 years	Herniated disc	Dull pain in the right side of the spine when UP, relief in SUP.	IU: renal descent when UP. Renography: reduced renal plasma flow.	Right nephroptosis	Retained TX 1 year. Right nephropexy	1 month: IU when UP does not descend and renography improves flow. 3 months: no pain.
**Clark et al.** **[43]**	1 Female 25 years	Joint hyperlaxity, morning stiffness in the lumbar spine, hands and legs.	Constant dull pain in the abdomen and right side, radiating to the groin. 10 months postpartum.	PE: joint laxity, mobile abdominal mass from right mid to lower quadrant. IU: right renal descent greater than 2 vertebral bodies when standing upright.	Formerly psychosomatic disorder, intestinal pathology. Right nephroptosis.	Psychiatry. Conservative TX with strengthening exercises and use of elastic corset.	Less intense symptomatology
**Nanayakkara et al.** **[44]**	1 Female 28 years		Abdominal pain from right lower back to groin and vomiting.	PE: tender palpation of the right abdominal area. IU: lowering and ventral rotation of the right kidney when UP. Renogram: reduced renal function.	Right nephroptosis	For 10 years, psychiatric TX. Right nephropexy.	1 year after nephropexy: kidney well positioned and pain-free.
**Lim et al. [45]**	1 Female 43 years		Focal and palpable intermittent abdominal mass in the SUP but not when UP. No pain.	PE: no palpable abdominal mass. Abdominal rx: right kidney small, rounded and descended when UP.	Right nephroptosis	Observation and follow-up	

AQ: abdominal quadrant; UP: standing upright; SUP: supine position; PE: physical examination; IU: intravenous urogram; LT: laboratory test; VAS: visual analogue scale; UPJO: ureteropelvic junction obstruction; Rx: radiography; CT: computed tomography; TX: treatment; Cm: centimetre.

**Table 2:** Pre-intervention kidney position and anatomical postural data.

STUDIES	SUPINE DECUBITUS						STANDING						CSS
	RP	UPK	VI	LHR	HIC	FC/PA	RP	UPK	VI	LHR	HIC	FC/PA	
**Khan et al. [27]**							L4		L2-3-4-5		↑	YES/ YES	
**Machado Bairo et al. [28]**	L2		L3-4			YES/ YES	L4		L2-3-4-5			YES/ YES	YES
**Lezrek et al. [29]**							L4-5	L3-4	T12 L-1-2-3-4-5	↓	↑	YES/ YES	
**O’Reilly et al. [30]**	L2	L1	L3-4-5	↓	↑	YES/ YES							
**Marcovich et al. [31]**	L2-3	L1	L3-4-5			YES/ YES	L5	L3-4	L1-2-3-4-5		↑	YES/ YES	YES
**Leong et al. [32]**							L2-3		L1-2-3-4-5	↓	↑	YES/ YES	
**Jungling et al. [33]** **Case 1**	L2-3	L1				NO/NO		L2-3	T12 L4	↓		YES/ YES	YES
**Jungling et al. [33]** **Case 2**	L3				↑	YES/ YES	S	S				NO/NO	YES
**Tunc et al. [34]**							L2-3	L1	T12 L2-3-4-5		↓	YES/ YES	
**Boylu et al. [35]**		L1	L3-4-5			YES/ YES	L4	L2-3	L3-4-5		↓	YES/ YES	YES
**Baldassarre et al. [36]**	L3-4	L3	L1-2-3-4-5		↑	YES/ YES	L5	L4	L2-3-4-5		↑	YES/ YES	YES
**Sood et al. [37]**	L3	L2	L2-3-5			YES/ YES	L4-5	L3-4	L2-3-4-5			YES/ YES	YES
**Wroclawski et al. [38]**	L2	L1	L1-2-3-4-5		↑	YES/ YES	L3-4	L2-3	L1-2-3-4-5	↑		NO/ YES	YES
**Hua Chan et al. [39]**	L2-3	L1	T12 L1-2-3-4		↓	YES/ YES	L4-5	L3	T12 L1-2-3-4-5	↓	↑	YES/ YES	YES
**Yoshida et al. [40]**	L2-3	L1					L4-5	L3	L1-2-3		↑	YES/ YES	YES
**Carola et al. [41]**							L3	L2	L1-2-3-4-5	↓		YES/ YES	
**Matsuda et al. [42]**									L1-2-3			YES/ YES	
**Clark et al. [43]**	L2-3	L1	L2-3-4	↓		YES/ YES	L4	L3	L1-2-3-4-5	↓		YES/ YES	YES
**Nanayakkara et al. [44]**	L2-3	L1	L3-4-5		↑	YES/ YES	L4	L3	L3-4-5		↑	YES/ YES	YES
**Lim et al. [45]**		L2	T12 L1-2-3			YES/ YES		L3-4	LDV T12 L1-2-3-4-5	↓	↑	YES/ YES	YES

RP: renal pelvis; UPK: upper pole kidney; VI: vertebrae involved; LHR: last homolateral rib; HIC: homolateral iliac crest; FC: flank closure; PA: protective attitude; S: sacrum; LDV: lower dorsal vertebrae; CSS: change from supine to standing; ↓: descended; ↑: ascended.

**Table 3:** Post-intervention kidney position and anatomical postural data.

STUDIES	SUPINE DECUBITUS							STANDING							CSS
	RP	UPK	VI	LHR	HIC	FC/PA	PPC	RP	UPK	VI	LHR	HIC	FC/PA	PPC	
**Khan et al. [27]**		L2	T12 L1-2-3			NO/ YES		L2		L2-3-4		↑	YES/ YES	YES	YES
**Machado Bairo et al. [28]**								L2		L2-3-4-5	↓		YES/ YES	YES	
**Lezrek et al. [29]**								L3-4	L2	L3-4-5	↓	↑	YES/ YES	YES	
**O’Reilly et al. [30]**															
**Marcovich et al. [31]**		L2	L3-4-5		↑	YES/ YES	YES		L3	L1-2-3-4-5		↑	YES/ YES	YES	NO
**Leong et al. [32]**															
**Jungling et al. [33]** **Case 1**								L2					YES/ YES	YES	
**Jungling et al. [33]** **Case 2**								L3-4	L2	L4-5		↑	YES/ YES	YES	
**Tunc et al. [34]**								L1-2	T12 L1	T12 L1-2-3-4-5		↓	YES/ YES	YES	
**Boylu et al. [35]**															
**Baldassarre et al. [36]**															
**Sood et al. [37]**															
**Wroclawski et al. [38]**															
**Hua Chan et al. [39]**															
**Yoshida et al. [40]**															
**Carola et al. [41]**															
**Matsuda et al. [42]**								L2-3		L1-2-3			YES/ YES	YES	
**Clark et al. [43]**															
**Nanayakkara et al. [44]**															
**Lim et al. [45]**															

RP: renal pelvis; UPK: upper pole kidney; VI: vertebrae involved; LHR: last homolateral rib; HIC: homolateral iliac crest; FC: flank closure; PA: protective attitude; S: sacrum; LDV: lower dorsal vertebrae; CSS: change from supine to standing; PPC: pre/post-intervention change; ↓: descended; ↑: ascended.

## Data Availability

Not applicable.

## Appendix A. CARE Checklist Results

**TOPIC**	**ITEM**	**STUDIES**					
		**Khan et al.** **[27]**	**Machado Bairo et al. [28]**	**Lezrek et al.** **[29] **	**O’Reilly et al.** **[30]**	**Marcovich et al. ** **[31]**	**Leong et al.** **[32]**
**TITLE**	**1**	NO	NO	NO	NO	NO	NO
KEYWORDS	**2**	YES	YES	YES	NO	NO	NO
ABSTRACT	**3a**	YES	YES	YES	YES	YES	NO
	**3b**	NO	NO	NO	YES	YES	NO
	**3c**	YES	YES	YES	NO	YES	NO
	**3d**	YES	YES	YES	NO	YES	NO
INTRODUCTION	**4**	YES	YES	YES	YES	NO	YES
PATIENT INFORMATION	**5a**	YES	YES	YES	YES	YES	YES
	**5b**	YES	YES	YES	YES	YES	YES
	**5c**	NO	NO	NO	NO	NO	NO
	**5d**	YES	YES	NO	YES	YES	NO
CLINICAL FINDINGS	**6**	YES	NO	YES	YES	YES	YES
TIMELINE	**7**	NO	NO	NO	NO	NO	NO
DIAGNOSTIC ASSESSMENT	**8a**	YES	YES	YES	YES	YES	YES
	**8b**	NO	NO	NO	NO	NO	NO
	**8c**	NO	NO	NO	NO	NO	NO
	**8d**	NO	NO	NO	NO	NO	NO
THERAPEUTIC INTERVENTION	**9a**	YES	YES	YES	YES	YES	YES
	**9b**	YES	YES	YES	NO	YES	NO
	**9c**	NO	NO	NO	NO	NO	NO
FOLLOW-UP AND OUTCOMES	**10a**	YES	YES	YES	YES	YES	NO
	**10b**	YES	YES	YES	YES	YES	YES
	**10c**	NO	NO	NO	NO	NO	NO
	**10d**	NO	NO	NO	NO	NO	NO
DISCUSSION	**11a**	YES	YES	YES	NO	YES	NO
	**11b**	YES	YES	YES	YES	YES	NO
	**11c**	YES	YES	YES	YES	YES	NO
	**11d**	YES	YES	YES	YES	YES	NO
PATIENT PERSPECTIVE	**12**	NO	NO	NO	NO	NO	NO
INFORMED CONSENT	**13**	NO	NO	NO	NO	NO	NO
	**TOTAL**	18/30	17/30	17/30	14/30	17/30	7/30

**TOPIC**	**ITEM**	**STUDIES**						
		**Jungling et al. 1º ** **[33]**	**Jungling et al. 2º** **[33]**	**Tunc et al.** **[34]**	**Boylu et al.** **[35]**	**Baldassarre et al.** **[36]**	**Sood et al.** **[37]**	**Wroclawski et al.** **[38]**
**TITLE**	**1**	NO	NO	NO	NO	NO	NO	NO
KEYWORDS	**2**	NO	NO	YES	NO	YES	NO	NO
ABSTRACT	**3a**	NO	NO	YES	YES	YES	NO	YES
	**3b**	NO	NO	NO	YES	YES	NO	YES
	**3c**	NO	NO	YES	YES	YES	NO	YES
	**3d**	NO	NO	YES	YES	YES	NO	YES
INTRODUCTION	**4**	YES	YES	YES	NO	YES	YES	NO
PATIENT INFORMATION	**5a**	YES	YES	YES	YES	YES	YES	NO
	**5b**	YES	YES	YES	YES	YES	YES	NO
	**5c**	YES	NO	NO	NO	NO	NO	NO
	**5d**	YES	NO	NO	NO	YES	NO	NO
CLINICAL FINDINGS	**6**	YES	YES	YES	YES	YES	YES	NO
TIMELINE	**7**	NO	YES	NO	NO	NO	NO	NO
DIAGNOSTIC ASSESSMENT	**8a**	YES	NO	YES	YES	YES	YES	YES
	**8b**	NO	NO	NO	NO	NO	NO	NO
	**8c**	NO	NO	NO	NO	NO	NO	NO
	**8d**	NO	NO	NO	NO	NO	NO	NO
THERAPEUTIC INTERVENTION	**9a**	YES	YES	YES	YES	YES	YES	NO
	**9b**	NO	NO	YES	YES	YES	NO	NO
	**9c**	NO	NO	NO	NO	NO	NO	NO
FOLLOW-UP AND OUTCOMES	**10a**	YES	YES	YES	YES	YES	YES	NO
	**10b**	YES	YES	YES	YES	YES	NO	NO
	**10c**	NO	NO	NO	NO	NO	NO	NO
	**10d**	NO	NO	NO	NO	NO	NO	NO
DISCUSSION	**11a**	NO	NO	YES	YES	NO	NO	NO
	**11b**	YES	YES	YES	YES	NO	NO	NO
	**11c**	YES	YES	YES	YES	YES	NO	NO
	**11d**	YES	YES	YES	YES	YES	NO	NO
PATIENT PERSPECTIVE	**12**	NO	NO	NO	NO	NO	NO	NO
INFORMED CONSENT	**13**	NO	NO	NO	NO	NO	NO	NO
	**TOTAL**	13/30	11/30	17/30	16/30	17/30	7/30	5/30

**TOPIC**	**ITEM**	**STUDIES**						
		**Hua Chan et al. [39]**	**Yoshida et al.** **[40]**	**Carola et al.** **[41]**	**Matsuda et al.** **[42]**	**Clark et al.** **[43]**	**Nanayakkara et al.** **[44]**	**Lim et al.** **[45]**
**TITLE**	**1**	NO	NO	NO	YES	NO	NO	NO
KEYWORDS	**2**	NO	YES	YES	YES	NO	NO	NO
ABSTRACT	**3a**	NO	NO	NO	NO	YES	NO	NO
	**3b**	NO	YES	NO	NO	YES	NO	NO
	**3c**	NO	YES	NO	NO	YES	NO	NO
	**3d**	NO	YES	NO	NO	YES	NO	NO
INTRODUCTION	**4**	NO	YES	NO	YES	YES	NO	NO
PATIENT INFORMATION	**5a**	YES	YES	YES	YES	YES	YES	YES
	**5b**	YES	YES	YES	YES	YES	YES	YES
	**5c**	YES	YES	NO	NO	NO	NO	NO
	**5d**	NO	NO	NO	NO	YES	NO	NO
CLINICAL FINDINGS	**6**	YES	YES	NO	NO	YES	YES	YES
TIMELINE	**7**	NO	YES	YES	NO	NO	NO	NO
DIAGNOSTIC ASSESSMENT	**8a**	YES	YES	YES	YES	YES	YES	YES
	**8b**	NO	NO	NO	NO	YES	YES	NO
	**8c**	YES	YES	NO	NO	NO	NO	YES
	**8d**	NO	NO	NO	NO	NO	NO	NO
THERAPEUTIC INTERVENTION	**9a**	YES	NO	NO	YES	YES	YES	YES
	**9b**	NO	NO	NO	YES	YES	NO	NO
	**9c**	NO	NO	NO	NO	NO	NO	NO
FOLLOW-UP AND OUTCOMES	**10a**	NO	NO	NO	YES	YES	YES	NO
	**10b**	NO	NO	NO	YES	YES	YES	NO
	**10c**	NO	NO	NO	NO	NO	NO	NO
	**10d**	NO	NO	NO	NO	NO	NO	NO
DISCUSSION	**11a**	NO	NO	NO	YES	NO	NO	NO
	**11b**	NO	YES	NO	YES	NO	NO	NO
	**11c**	NO	YES	NO	YES	YES	NO	NO
	**11d**	NO	YES	NO	YES	YES	NO	NO
PATIENT PERSPECTIVE	**12**	NO	NO	NO	NO	NO	NO	NO
INFORMED CONSENT	**13**	NO	YES	NO	NO	NO	NO	NO
	**TOTAL**	7/30	16/30	5/30	14/30	17/30	8/30	6/30
